# The Nottingham Prognostic Index: 5-year and 10-year survival data for all-cause survival within a screened population

**DOI:** 10.1186/bcr3274

**Published:** 2012-11-09

**Authors:** YF Fong, J Evans, D Brookes, K Gower Thomas

**Affiliations:** 1 Royal Glamorgan Hospital, Llantrisant, UK; 2 Breast Test Wales, Cardiff, UK

## Introduction

The Nottingham Prognostic Index (NPI) is an accepted prognostication tool in the management of breast cancers. The latest overall 5-year and 10-year breast cancer survival has been reported to be 85% [1] and 77% [2]. We applied the NPI to breast cancers diagnosed within Breast Test Wales and present survival data in each NPI category.

## Methods

All women with screen-detected and interval cancers having had screening between 1998 and 2001 were included. The NPI for each cancer was calculated with the size, nodal status and grade of the primary tumour. Survival data (all cause) was calculated after 10 years of follow-up.

## Results

In the 3-year screening period, 199,082 women were screened. A total of 1,712 cancers were diagnosed. In total, 1,546 had data available for calculating the NPI. The overall 5-year and 10-year survival was 94% and 82%. See Table 1.

**Table 1:** 

NPI category	Number of cases	5-year survival (%)	10-year survival (%)
1	322 (20.8%)	97	89
2	537 (34.7%)	93	84
3	536 (34.7%)	90	77
4	151 (9.8%)	88	73

## Conclusion

The overall 5-year and 10-year survival (all cause) has improved even when compared with specific breast cancer survival of recent published data. Our data provide a reference for updating all-cause survival of women diagnosed with breast cancers within a screened population.
